# Assessment of the Effectiveness of Educating Elderly Residents in an Urban Community in South India on Managing Post-COVID-19 Musculoskeletal Complications

**DOI:** 10.7759/cureus.70857

**Published:** 2024-10-04

**Authors:** Naga Prasanna Kumari Balisetty, A. Maria Therese, Abhilasha Rao, ​P.D. Divya, K.K. Shiny John

**Affiliations:** 1 Medical Surgical Nursing, Eashwari Bai Memorial College of Nursing, Secunderabad, IND; 2 Medical Surgical Nursing, Mother Theresa Post Graduate and Research Institute of Health Sciences, Puducherry, IND; 3 Nursing, Eashwari Bai Memorial College of Nursing, Secunderabad, IND; 4 Community Health Nursing, Eashwari Bai Memorial College of Nursing, Secunderabad, IND

**Keywords:** complications, covid-19, elderly patients, musculoskeletal complications, nursing, post-covid-19 complications

## Abstract

Objective

This study aimed to enhance post-COVID-19 patients’ understanding of musculoskeletal issues and their management through a structured educational program in the elderly population.

Methodology

The study included 60 participants. Each participant was assessed using sociodemographic tools and a structured knowledge questionnaire on post-COVID-19 musculoskeletal complications among elderly individuals. A pretest was conducted on all participants using these tools. Subsequently, a seven-day structured training program was administered, followed by a post-test using the same tools. A comparative analysis was performed to evaluate the difference in understanding between the pretest and post-test results.

Results

The pretest comprehension levels in the below-average and average groups showed a mean and standard deviation of 3±1.5 and 14.5±2.0, respectively, with a statistically significant p-value of 0.0001 and a t-value of 15.6. In the post-test, proficiency levels in the average and above-average groups demonstrated a mean and standard deviation of 18.2±1.5 and 24.5±3.6, respectively, with a statistically significant t-value of 6.88 and a p-value of 0.0001. Among the elderly participants, there was a substantial improvement in competence between the pre-and post-test scores, with a highly significant p-value of 0.0001.

Conclusion

Our study concluded that the elderly population was particularly vulnerable to various diseases and complications due to aging, making it essential to provide education on managing post-COVID-19 musculoskeletal issues.

## Introduction

The COVID-19 pandemic stands as the most significant global threat since World War II, marking the onset of a worldwide health crisis. The human population faced an overwhelming burden of grief as the death toll exceeded two million [1]. This disease first emerged in Wuhan, China, in December 2019 and quickly spread to every continent except Antarctica [2]. According to the Post-COVID Functional Scale (PCFS), most individuals who recovered from COVID-19 experienced various functional impairments, ranging from mild to severe. This finding is highlighted by research on the functional state of post-COVID patients and their associated factors [3].

The characteristics of post-acute COVID-19 can vary significantly. Even a seemingly mild case of COVID-19 can result in persistent symptoms, including fatigue, low-grade fever, and recurrent cough, which may fluctuate over time [4]. Other reported symptoms include respiratory difficulties, headaches, swallowing problems, neurocognitive issues, rashes, gastrointestinal disturbances, metabolic irregularities (such as uncontrolled diabetes), thromboembolic conditions, depression, and other mental health concerns [5]. Skin rashes may manifest as vesicular, maculopapular, urticarial, or chilblain-like lesions on the extremities (commonly known as “COVID toe”) [6]. However, these typically do not warrant referral or further investigation if the patient is otherwise in excellent health [7].

While musculoskeletal issues have been frequently reported during COVID-19 [8], the spectrum of long-term consequences on the musculoskeletal system remains under-documented. Initial mild complaints often include persistent fatigue and restlessness, which can escalate to more severe symptoms such as myalgia, joint pain, and arthritic flare-ups [9]. Case reports have also documented acute instances where previously stable elderly individuals experienced significant complications such as fractures [10], hematomas [11], and even spinal abscesses after recovering from COVID-19 [12]. These severe cases highlight the varied impact of the virus on musculoskeletal health. Furthermore, the extensive use of steroids, a cornerstone of life-saving treatments during the pandemic, has exacerbated musculoskeletal issues. Their well-documented adverse effects on bone density and muscle strength, combined with their widespread availability over the counter, pose additional risks for the elderly [13]. Clinical observations continue to underscore the prevalence of these post-COVID-19 musculoskeletal complications, revealing a critical gap in patient awareness and management of these conditions [14].

Elderly individuals who have suffered from COVID-19 often report persistent or acute restlessness, myalgia, joint pain, arthritic conditions, and reduced muscle strength [9]. The well-documented adverse effects of steroids on the musculoskeletal system, coupled with their widespread over-the-counter availability, are of particular concern, given that steroids were a cornerstone of life-saving treatments during the pandemic [13]. Clinical observations indicate that post-COVID-19 musculoskeletal complications are increasingly prevalent, with many patients lacking awareness of these complications and how to manage them [14].

The elderly population is especially vulnerable to complications due to aging, making them more susceptible to adverse outcomes during and after the pandemic. Various studies have outlined why this demographic is at a greater risk, attributing it to factors such as frailty, pre-existing health conditions, and diminished immune response [15-17]. Understanding these vulnerabilities is essential in developing effective management and preventive strategies. Therefore, this study aimed to improve post-COVID-19 patients' understanding of musculoskeletal issues and their management through a structured educational program within a selected community.

## Materials and methods

Study design and participants

This study employed a one-group pretest-posttest design, a pre-experimental methodology, to evaluate the effectiveness of an educational intervention. It involved 60 elderly individuals diagnosed with post-COVID-19 musculoskeletal complications, recruited over two weeks from September 19, 2023, to September 26, 2023, after receiving ethical approval from Eashwaribai Memorial College of Nursing, Hyderabad, Telangana, India (approval reference number: EBMCON/IEC/2022/002). Participants were selected using convenience sampling, a non-probability method. All participants had experienced musculoskeletal issues related to COVID-19. Each participant received a thorough introduction, during which the researcher explained the study’s purpose and confirmed their willingness to participate. All necessary measures were taken to protect the privacy and confidentiality of participants’ personal information. The study adhered to the principles of the Declaration of Helsinki, and informed consent was obtained from all participants. 

Inclusion and exclusion criteria

The study included participants aged 60 years or older, available during data collection, and able to communicate in Telugu or English. Both male and female participants were included, provided they consented to participate. Exclusion criteria included individuals younger than 60 years, those uninterested in participating, and those with linguistic barriers.

Data collection tools

The study used a sociodemographic tool with two parts. Part I covered demographic factors such as age, gender, education, religion, diet, vaccination status against COVID-19, incidence of post-COVID musculoskeletal complications, previous knowledge of COVID-19, and sources of information. A rating key was generated by coding these factors. Part II consisted of a structured questionnaire focused on the management of post-COVID-19 musculoskeletal complications. Responses in part II were rated on a scale, with 0 indicating an incorrect response and 1 indicating a correct response, for a maximum possible score of 30. Understanding levels were categorized as minimal (1-10), moderate (11-20), and strong (21-30).

Pretest data were collected from the participants using the described tools. A well-designed survey was distributed to assess their understanding of managing post-COVID-19 challenges. An educational intervention, including multimedia tools and a resource leaflet on the prevention and management of COVID-19, was delivered by experts in the field of nursing. The effectiveness of this educational program was assessed using a post-test conducted on the seventh day using the same instruments.

Tool development

A structured understanding survey was employed to gauge the elderly population’s familiarity with post-COVID-19 musculoskeletal issues. This tool was developed after a thorough literature review and consultations with subject matter experts, ensuring its relevance and effectiveness. The initial draft of the tool contained 40 items, which were refined based on expert recommendations, resulting in a final version with 30 items. Pretest data were gathered using planned interviews and the knowledge survey. The educational program was found to be effective based on the participants’ improved understanding in the post-test.

Statistical analysis

Data collection involved the systematic gathering and organization of empirical data to analyze the research hypothesis. Both inferential and descriptive statistics were used in data analysis and interpretation. Data were organized, coded, and entered into an Excel sheet before analysis with SPSS Version 20 (IBM Corp., Armonk, NY). A p-value of 0.05 or less was considered statistically significant. The frequency and proportional breakdown of each demographic variable were calculated. The Welch t-test was used to assess pre- and post-test comprehension scores. An unpaired t-test was used to compare outcomes before and after the intervention. The correlation between post-test knowledge scores and specific demographic factors was analyzed using a chi-square test.

## Results

The study encompassed a diverse demographic, with age groups predominantly represented by those aged 70-75 and above 75 years, each constituting 20 (33.3%) of the participants. The gender distribution showed 34 (56.67%) males and 26 (43.3%) females, with no representation of other gender identities. The participants' educational qualifications varied, with 28 (46.6%) having a secondary education, 12 (20%) having a primary education, and 10 (16.6%) each possessing graduation or other qualifications. A total of 23 participants (38.3%) identified as Hindu, 22 (36.6%) as Muslim, and 15 (25%) as Christian. Dietary habits leaned toward non-vegetarian preferences in 34 participants (56.6%). All participants, comprising 47 (78.3%), had previously contracted COVID-19 and had received full vaccinations against the virus. Prior knowledge about the topic was limited, with only eight (13.3%) participants having any awareness, predominantly sourced from health professionals (20 participants, 33.3%) and social media (13 participants, 21.7%) (Table 1).

**Table 1:** Frequency and proportion distribution of elderly folks in accordance with historical variables

Background variables	Frequency, N=60	Percentage
Age groups		
60-65 years	10	16.6%
65-70 years	10	16.6%
70-75 years	20	33.3%
Above 75 years	20	33.3%
Gender		
Male	34	56.67%
Female	26	43.3%
Transgender	0	0%
Educational qualification		
Primary education	12	20%
Secondary education	28	46.6%
Graduation	10	16.6%
Others	10	16.6%
Religion		
Hindu	23	38.3%
Muslim	22	36.6%
Christian	15	25%
Others	0	0%
Type of diet		
Vegetarian	26	43.3%
Non-vegetarian	34	56.6%
Did you ever get infected with COVID-19		
Yes	47	78.3%
No	13	21.6%
Are you completely vaccinated against COVID-19		
Yes	60	100%
No	0	0%
Previous knowledge		
Yes	8	13.3%
No	52	86.6%
Source of information		
Social media	14	23.3%
Peer group	13	21.6%
Health professional	20	33.3%
Mass media	13	21.6%

The pre-test results, categorized by performance levels, demonstrated a significant difference in mean scores across the groups. Participants in the below-average group (n=52) had a mean score of 3 with a standard deviation of 1.5. A t-test revealed a highly significant p-value of 0.0001, indicating that the observed difference was statistically significant at the 95% confidence interval, with a t-value of 15.6 and 8 degrees of freedom (df). In contrast, the average group (n=8) showed a higher mean score of 14.5 with a standard deviation of 2.0. No participants were classified in the above-average group, resulting in a mean score of 0 for that category (Table 2).

**Table 2:** Average score of the pre- and post-test groups Data have been presented as mean ± standard deviation Both p-values show significance

Groups	Below average	Average	Above average	Student t-value	p-Value
Pre-test	3 ± 1.5	14.5 ± 2	0 ± 0	15.6	0.0001
Post-test	0 ± 0	18.2 ± 1.5	24.5 ± 3.6	6.88	0.0001

**Figure 1:**
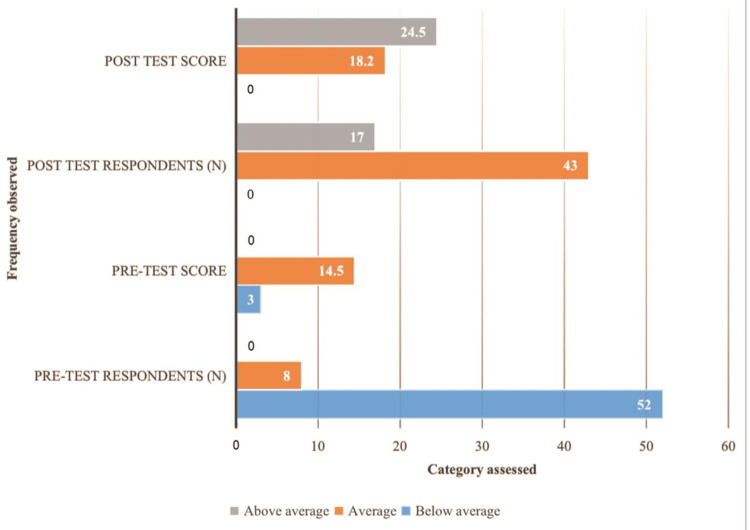
Pre- and post-test scores Participants were divided into three groups: below average, average, and above average. In the pre-test, no participants scored in the above average group, and thus the number of respondents and the pre-test score for this group were both 0. In the post-test, no participants scored in the below-average group, and thus the number of respondents and the post-test score for this group were also 0. Therefore, the zeros in the pre-test and post-test scores indicate this.

These results highlight a clear disparity between the below-average and average groups, emphasizing the statistical significance of the findings. The post-test results, categorized by performance levels, revealed a significant difference in the mean scores across the groups. No participants were classified in the below-average group, resulting in a mean score of 0. A t-test yielded a highly significant p-value of 0.0001, indicating statistical significance at the 95% confidence interval, with a t-value of 6.88 and 19 df. The average group (n=43) had a mean score of 18.2 with a standard deviation of 1.5. Meanwhile, the above-average group (n=17) demonstrated the highest mean score of 24.5 with a standard deviation of 3.6. These findings highlight the statistically significant differences between the groups, with a notable increase in performance in the average and above-average categories (Figure 1).

The comparison between the pre- and post-test demonstrates a significant improvement. The mean score increased from 5.5 in the pre-test to 20.8 in the post-test, resulting in a mean difference of 15.3. The calculated t-value of 14.0871 and the p-value of less than 0.0001 indicate that this improvement is statistically significant at the 95% confidence interval, with 102 df. These results suggest that the intervention enhanced the participants’ knowledge (Table 3).

**Table 3:** Comparison of older individuals' pre- and post-test expertise in handling musculoskeletal problems following COVID-19 Student t-test was performed A p-value of <0.05 was considered significant

Area of knowledge	Pre-test mean	Post-test mean	Mean difference	t-Value	p-Value
Overall knowledge scores	5.5	20.8	15.3	14.0871	<0.0001

A substantial difference was observed among age categories, with a p-value of 0.0001 at a significance level of less than 0.05. At an acceptable level of significance of <0.05, no statistically significant difference was found between the demographic variables of gender, educational status, religion, information source, and previous COVID-19 infection (p values of 0.429, 0.684, 0.083, 0.449, and 0.241, respectively). However, at the significance threshold of less than 0.05, a significant difference was found in dietary preferences among elderly attendees, with a p-value of 0.035 (Table 4).

**Table 4:** Association between elderly people's post-test understanding scores and specific socioeconomic factors on the management of post-COVID-19 musculoskeletal issues The chi-square test was performed A p-value of <0.05 was considered significant

Variables	Below average	Average	Above average	Chi-square statistic	p-Value
Age groups (in years)					
60-65	0%	30%	70%	25.77	0.00001
66-70	0%	30%	70%		
71-75	0%	95%	5%		
>75	0%	90%	10%		
Gender					
Male	0%	67.6%	32.3%	0.624	0.429
Female	0%	76.9%	23.06%		
Educational status					
Illiterate	0%	66.6%	33.3%	1.4892	0.684
Primary education	0%	78.5%	21.4%		
Secondary education	0%	70%	30%		
Graduate and above	0%	60%	40%		
Religion					
Hindu	0%	56.5%	43.4%	4.958	0.083
Muslim	0%	86.3%	13.6%		
Christian	0%	73.3%	26.6%		
Diet					
Vegetarian	0%	57.6%	42.3%	4.412	0.0356
Non-vegetarian	0%	82.3%	17.6%		
Did you ever get infected with COVID-19					
Yes	0%	68%	31.9%	1.370	0.241
No	0%	84.6%	15.3%		
Source of information about the post-COVID-19 musculoskeletal complication					
Social media	0%	78.5%	21.4%	2.648	0.449
Peer group	0%	76.9%	23%		
Mass media	0%	75%	25%		
Medical personnel	0%	53.8%	46.1%		

## Discussion

As the pandemic continues to spread, it is increasingly clear that many survivors endure a range of illnesses even after recovering from the initial infection. Research conducted by the Washington University School of Medicine in St. Louis indicates that individuals who recover from COVID-19, including those who were not severely ill or hospitalized, face a heightened risk of unexpected death within six months of their diagnosis. The research team has compiled an extensive catalog of diseases associated with COVID-19, providing a comprehensive overview of the virus’s potential long-term consequences [18].

Globally, the pandemic has had a detrimental impact on healthcare systems and has imposed significant social and economic burdens. The official death toll has surpassed six million since the pandemic began [1]. Initially, the extent of COVID-19’s multisystem involvement was unclear. While the virus was initially believed to primarily affect the lungs, it is now known that COVID-19 has a wide spectrum of clinical implications. Growing evidence indicates that COVID-19 affects neurological, musculoskeletal, cardiovascular, and gastrointestinal systems, either directly or indirectly, leading to a variety of symptoms [19].

In India, more than 18.7 million confirmed COVID-19 cases have been reported, with infections continuing to rise across South Asia. In the southern state of Kerala alone, there have been 1,533,984 confirmed cases. The positivity rate is alarmingly high, at 25% above the national average. The recovery rate stands at 81.2%, with 1,244,300 recoveries, while the death toll has reached 55,295 [20].

Our study found no significant differences between genders, likely due to the relatively balanced number of male and female participants. However, we observed notable differences among age groups. Consistent with the research conducted by Cevei et al., older adults are particularly vulnerable to COVID-19 complications, especially those with pre-existing conditions [15]. Steinmeyer et al. also emphasize that older adults are a highly diverse group, and assessing individual impairments using the CIRS-G scale is crucial. They argue that closely monitoring older patients’ health and conducting multidisciplinary assessments is vital for identifying those with chronic conditions at the highest risk of adverse health outcomes [16].

Similarly, Moloney et al. [17] assessed the overall comorbidity burden in older adults using the CIRS-G scale and found that more than one-third of participants scored 108 or higher. It is important to recognize that age does not always correlate with an individual’s ability to recover from severe health events [21]. We also found significant dietary differences among elderly participants. Research by Damayanthi and Prabani reported that malnutrition is particularly prevalent among older adults with COVID-19, predicting unfavorable outcomes, including hospitalization and admission to intensive care units [22].

In our study, the knowledge levels among elderly participants during the pre-test were primarily below average and average (p-value of 0.0001). However, in the post-test, knowledge levels improved to mostly average and above average (p-value of 0.0001). Our findings showed a statistically significant difference between pre-test and post-test results (p-value < 0.0001), consistent with a study by Karhade et al. [23]. Their study found that 16.67% of respondents initially had limited knowledge, 50% had moderate knowledge, and 33.33% had high knowledge [23]. None of the respondents achieved very high competence levels in the pre-test. In contrast, post-test results showed that 48.33% of participants scored at a very high level of competence, 5% at an excellent level, and 46.67% at a good level, with no participants receiving low or mediocre scores [23].

Limitations

This study had several limitations. First, the sample size was relatively small, comprising only 60 participants, which may limit the generalizability of the findings to a broader population. Additionally, the study was conducted within a single community, which may not represent the diverse experiences and needs of elderly individuals in other regions. The study also relied on self-reported data, which could be subject to bias or inaccuracies. Moreover, the short duration of the structured educational program may not have been sufficient to ensure long-term retention of the information provided. Finally, the study did not include a control group, which would have allowed for a more rigorous comparison of the effectiveness of the intervention.

## Conclusions

Our study concluded that the elderly population was particularly vulnerable to various diseases and complications due to aging, making it essential to provide education on managing post-COVID-19 musculoskeletal issues. Effective education enabled older adults to implement appropriate preventive and control measures, thereby improving their quality of life. The investigation revealed a correlation between knowledge levels and understanding of COVID-19-related issues, which was influenced by factors such as age, educational attainment, and diet. The study demonstrated that tailored educational sessions focusing on post-COVID-19 issues were effective as a teaching strategy for specific populations. Future research should explore the long-term sustainability of educational interventions on elderly populations, focusing on retention of knowledge and behavior modification. Additionally, there is a need to investigate the efficacy of similar programs in larger and more diverse populations, as well as their potential for addressing other post-COVID complications. This study could also inform the development of public health policies aimed at enhancing elderly care, particularly in resource-limited settings where access to health education is restricted.
